# How aging may impact the failure to rescue after colorectal laparoscopic surgery. Analysis of 1000 patients in a single high-volume center

**DOI:** 10.1007/s13304-025-02173-6

**Published:** 2025-03-31

**Authors:** Rosa Marcellinaro, Aldo Rocca, Pasquale Avella, Michele Grieco, Domenico Spoletini, Massimo Carlini

**Affiliations:** 1 Department of General Surgery, General Surgery Unit, S. Eugenio Hospital, Rome, Italy; 2 Department of Medicine and Health Science, University of Molise, Campobasso, Italy; 3 Hepatobiliary and Pancreatic Unit, Pineta Grande Hospital, Castel Volturno, Italy; 4 Department of Clinical Medicine and Surgery, University of Naples “Federico II”, Naples, Italy

**Keywords:** Failure to rescue, Colorectal surgery, Frailty, Elderly, Minimally invasive surgery, Laparoscopy, Complications

## Abstract

This study aimed to evaluate the FTR after laparoscopic colorectal surgery in an Italian high-volume centre. A retrospective analysis was conducted in a consecutive series of patients who underwent elective laparoscopic colorectal surgery for neoplastic disease between January 2010 and December 2023 at the General Surgery Department of the San Eugenio Hospital, Rome, Italy. Patients were grouped by age in adult (vs. < 75 years) and elderly group (≥ 75 years). A multivariate analysis of the predictive factors of complications was performed. A total of 1,000 patients met the inclusion criteria, excluding those who underwent open or robotic surgery, either in emergency or elective settings. 53 patients (5.3%) experienced major complications. The mean age of patients with no or mild complications was 65.60 years (± 10.61), whereas patients with severe complications were older (69.94 years ± 12.02, *p* = *0.0041*). Gender distribution and BMI do not represent a risk factor for major complications (*p* = *0.2555* and *p* = *0.2686*, respectively), unlike the ASA score III or IV (*p* = *0.0001*). The overall FTR rate for adult patients is 9%, while it is slightly higher at 10% for elderly patients. No statistical differences were found between the 2 groups. Elderly patients had more frequent FTR due to infective complications, while the FTR rate for cardiovascular disease was more frequent in the adult group. Minimally invasive approach, skilled team, well-established rapid response and standardized complication management protocols can positively impact FTR regardless of patients' age.

## Introduction

Colorectal surgery is afflicted by a non-negligible percentage of complications and the mortality rate varies from 1 to 25% according to recent literature [1–7]. Improvements in surgical techniques and technologies and some initiatives like surgical safety checklist and early warning scores have contributed to reducing postoperative mortality after colorectal surgery [8]. Minimally invasive surgery played a fundamental role in this trend [9–15]. At the same time, there has been a deep change in the perioperative management of patients through careful administration of antibiotics and mechanical intestinal preparation and ensuring the biodiversity of the intestinal microbiota [16, 17]. Moreover, the perioperative management of patients according to the ERAS principles has favored an improvement in terms of surgical outcomes with a lower incidence of postoperative complications [18]. This approach has also proven successful in fragile patients, especially the elderly ones who benefit most from it [19].

The elderly are defined as having a chronologic age of 65 years or older. In 2016, persons defined elderly constituted 19.2% of the European population and this number is still growing. However, most people aged 65 or over are still able to work and lead an active life. Many recent studies suggest moving the cut off by 10 years and considering as elderly those aged 75 or over [20].

Age is considered an important factor in predicting postoperative morbidity, mortality, and failure to rescue (FTR) after surgery [21–26]. Furthermore, frailty — a clinically identifiable state of heightened vulnerability, arising from an aging-related decline in physiologic reserve and function across multiple organ systems — represents a common syndrome linked to increased risk for poor health outcomes including falls, incident disability, hospitalization, and mortality [22, 24, 27, 28].

FTR is defined as the rate of death following postoperative major complications and it reflects the ability to rescue a patient from the risk of death [29]. This parameter was introduced first in 1992 and currently reflects the quality of care during hospitalization and appears to be related to patient characteristic and hospital factors [30, 31].

Aim of this study was to evaluate the FTR after laparoscopic colorectal surgery in an Italian high-volume center.

## Methods

### Study design

This study aims to analyze and describe surgical outcomes of elderly patients (≥ 75 years), compared to the control series of adult patients (< 75 years), who underwent laparoscopic colorectal resection for neoplastic disease. At the General Surgery Department of the San Eugenio Hospital, Rome, Italy, from January 2010 to December 2023, 1277 laparoscopic colorectal resections were performed. Out of these, the first 1000 patients who underwent colorectal resections for neoplastic disease were evaluated.

Demographic, clinical, surgical, and pathologic data were prospectively collected and retrospectively reviewed according to the Strengthening the Reporting of Observational Studies in Epidemiology (STROBE) statement [32].

All patients signed an informed written consent and Institutional Review Board approval by the University of Molise was waived according to the Declaration of Helsinki guidelines (protocol number 10/21, approved date: May 12, 2021). Patients ≥ 18 and < 75 years (adults) were considered as control group. All procedures were performed in elective surgery. One thousand patients were included.

### Endpoints

The primary outcome was to compare FTR between adults and elderly patients. The FTR-Surgical (FTR-S) index was defined as the percentage of patients with a surgical complication (anastomotic dehiscence, ileus, surgical site infections, colonic ischemia) who died within 30 days of surgery.

The FTR-Nonoperative (FTR-NonOp) was defined as 30-day mortality following a nonoperative complication within 30 days of surgery. Complications included ≥ 1 of acute kidney injury (AKI), cardiac arrest, cardiac arrhythmia, myocardial infarction, sepsis, shock, venous thromboembolism, pneumonia, respiratory failure, stroke, or delirium.

Secondary outcomes included pre-, intra-, and postoperative variables analysis to identify predictive factors involved in major complications.

### Data collection

The surgeons’ team compiled a Microsoft Excel Database, including demographic data such as age, sex, Body Mass Index (BMI), clinical variables as American Society of Anesthesiologist (ASA) score [33], comorbidities, type of disease, colon or rectum location and stage of malignancies according to AJCC Editions — Staging Criteria [34, 35], previous abdominal surgical or radiotherapy (RT) and/or chemotherapy (CHT), operative data as type of procedure and anastomosis (intracorporeal, extracorporeal or coloanal anastomosis), operative time (minutes), conversion to open surgery and postoperative outcomes.

Postoperative morbidities were classified according to the Clavien‒Dindo (CD) classification [36]. Severe surgical complications were defined as CD grade ≥ 3.

Clinically, radiologically, or endoscopically leak of luminal contents from a surgical join was categorized as anastomotic dehiscence [37].

### Pre-operative management

Pre-operative investigations including diagnosis and staging by colonoscopy with tattooing of the tumor and by a total body Computed Tomography (CT) scan according to Colorectal Cancer Italian Guidelines [38], in case of malignant diseases. In rectal cancer, patients also underwent Magnetic Resonance Imaging (MRI) to overcome CT limitations and to evaluate tumor location and morphology, T category, anal sphincter complex involvement, Circumferential Resection Margin (CRM) status, involvement of the pelvic sidewall, extramural vascular invasion (EMVI), and N category to select patient eligible to neoadjuvant RT and CHT [39–41].

All cases were discussed at the Multidisciplinary Team (MDT) for gastrointestinal tumor composed of oncologists, radiologists, anesthesiologists, nurses, pathologists and surgeons to define better patient’ management. Patients affected by concomitant extra-colonic and rectal tumors were excluded. According to the Enhance Recovery After Surgery (ERAS) protocol [17], a dedicated prehabilitation to improve the functional capability of patients before surgical procedure was provided. Perioperative low-molecular-weight heparin was administered in all patients.

In December 2020, the *MIRACLe* protocol was introduced in our clinical practice to implement the intestinal microbiota and reduce the incidence of anastomotic complications. This protocol consists of the administration of low-volume Mechanical Bowel Preparation (MBP) for all patients, regardless of the type of surgical resection; oral antibiotics only (intravenous antibiotics are abolished); oral preoperative, intraoperative intraluminal anastomotic and oral postoperative probiotics [16, 17, 42].

### Intra- and postoperative workup

The surgical procedures were subdivided into right colectomy, transverse resection, left flexure resection, left colectomy, sigmoidectomy, rectum resection, Hartmann procedure, abdominoperineal resection, total colectomy. Technologies, devices, surgical techniques and pre- and postoperative clinical practices were superimposable in both groups [10, 19, 43–47]. Colon and rectum resections were performed by the same surgical team tutored by a senior surgeon (M.C.).

In both adult and elderly patients, we followed the postoperative items of the ERAS protocol: bladder catheter removal and oral liquid administration on POD1. In the case of intraoperative nasogastric tube positioning, it was removed at the surgical procedure end or, in left flexure resections, on POD1. All uncomplicated patients were encouraged to mobilize from the first POD. Discharge was authorized if no postoperative complications, toleration of a normal diet without nausea or vomiting, good pain control, and restoration of flatus or stool passage were obtained [16].

### Statistical analysis

The quantitative data are reported as mean ± standard deviation (SD) and median (range). Normally distributed quantitative data were analyzed with a *t-*test. The qualitative data are reported as the number of patients (percentage) and a comparison was performed by Fisher’s exact test. All tests were two-sided with a significance level of 5%. Statistical significance was defined as a *p* value < 0.05.

The analyses were performed using IBM SPSS, version 23 (IBM Co., Armonk, NY, USA).

## Results

A total of 1000 patients underwent CRC laparoscopic surgery: 657 (65.7%) were younger than 75 years and 343 (34.3%) ≥ 75 years old. The mean age across all patients was 69.61 years (± 10.68), while 64.15 years (± 8.72) and 80.36 years (± 3.82) for < 75 years and ≥ 75 years groups respectively, with a statistically significant difference (*p* = *0.0001).*

Out of the total cohort, 443 patients (44.3%) were female: 283 (43.07%) in the younger group and 160 (46.65%) in the older group. The gender difference was not statistically significant (*p* = *0.2842*). According to age distribution, the baseline characteristics of patients are listed in Table 1**.**

**Table 1:** Study population and baseline characteristics of patients according to age groups

	All	< 75 years	≥ 75 years	*p* value
Number of patients; *n*. (%)	1000 (100)	657 (65.70)	343 (34.30)	
Age, years;				
Mean ± SD	69.61 ± 10.68	64.15 ± 8.72	80.36 ± 3.82	**0.0001**
Median (IQR)	71 (22–97)	66 (22–74)	80 (75–97)	
Female gender; *n*. (%)	443 (44.30)	283 (43.07)	160 (46.65)	0.2842
BMI; kg/m^2^;				
Mean ± SD	25.87 ± 4.51	25.93 ± 4.41	25.59 ± 4.99	0.2490
Median (IQR)	25.38 (14.51–44.44)	25.47 (14.69–43.76)	25.52 (14.51–44.44)	
ASA classification; *n*. (%)				
I	83 (8.30)	81 (12.33)	2 (0.58)	**0.0001**
II	690 (69)	473 (71.99)	217 (63.26)	
III	222 (22.20)	101 (15.37)	121 (35.28)	
IV	5 (0.5)	2 (0.31)	3 (0.88)	
Comorbidities; *n*. (%)				
Cardiac disease	246 (24.60)	164 (24.96)	82 (23.91)	0.7572
Respiratory disease	22 (2.20)	15 (2.28)	7 (2.04)	1.0000
Other	130 (13)	89 (13.55)	41 (11.95)	0.5524
Multiple diseases	136 (13.60)	68 (10.35)	68 (19.82)	0.0001
Benignant disease; *n*. (%)				
Colon	95 (9.50)	70 (10.66)	25 (7.29)	0.0893
Rectum	0	0	0	
Malignant disease; *n* (%)				
Colon	776 (77.60)	495 (75.24)	281 (81.92)	0.1110
Rectum	129 (12.90)	92 (14)	37 (10.79)	
Stage for malignant disease; *n* (%)				
Stage 1	237 (23.70)	165 (25.12)	72 (20.99)	0.0618
Stage 2	333 (33.30)	199 (30.28)	134 (39.08)	
Stage 3	266 (26.60)	174 (26.48)	92 (26.81)	
Stage 4	69 (6.90)	49 (7.46)	20 (5.83)	
Previous abdominal surgery; *n*. (%)	148 (14.80)	29 (4.41)	119 (34.69)	**0.0001**
Previous chemotherapy; *n*. (%)	5 (0.50)	4 (0.61)	1 (0.29)	0.6655
Previous radiotherapy; *n*. (%)	2 (0.20)	1 (0.15)	1 (0.29)	1.0000
Previous radio-chemotherapy; *n*. (%)	27 (2.70)	25 (3.80)	2 (0.58)	**0.0017**

Bold values indicate statistical differences

*BMI* Body Mass Index; *ASA score* American Society of Anaesthesiologists score

Previous abdominal surgery and neoadjuvant radio-chemotherapy rates were significantly higher in older patients. All patients experienced laparoscopic surgery and 23 out of 1000 patients (2.3%) required conversion from laparoscopic to open surgery: 14 (60.87%) in the adult cohort and 9 (39.13%) in elderly patients. The difference in conversion rates between the two age groups was not statistically significant (*p* = *0.6591*).

In the control group (> 75 years), conversion was required in 6 (42.86%) right hemicolectomies, 3 (21.43%) transverse colon resections, 2 (14.28%) left hemicolectomies, 2 (14.28%) sigmoidectomies, and 1 (7.15%) anterior rectal resection with total mesorectal excision. In the elderly group, conversion was necessary during 1 (11.11%) right hemicolectomy, 1 (11.11%) transverse colon resection, 2 (22.22%) splenic flexure resections, 2 (22.22%) left hemicolectomies, 1 (11.11%) sigmoid resection, 1 (11.11%) rectal resection with partial mesorectal excision, and in 1 (11.11%) case of multiple segmental colonic resections. In more than 60% of cases, the reasons for conversion were related to local tumor extension and the presence of peritoneal adhesions. However, most conversions in both groups were recorded at the beginning of the laparoscopic experience.

Concerning surgical techniques, the right colectomy was the most common procedure, performed in 426 patients (42.6% of all cases), with a higher rate in the elderly group (*p* = *0.0127*). Contrarily, the transverse colon resection was the less common procedure, with superimposable rates (3.9% and 4.08% in younger and older cohorts respectively, *p* = *0.8640*). These data are summarized in Table 2**.**

**Table 2:** Intra and postoperative outcomes of patients who underwent colorectal resection according to age groups

	All	< 75 years	≥ 75 years	*p* value
Number of patients; (%)	1000 (100)	657 (65.70)	343 (34.30)	
Conversion to open surgery, *n*. (%)	23 (2.3)	14 (2.13)	9 (2.62)	**0.6591**
Procedures; *n*. (%)				
Right colectomy	426 (42.60)	261 (39.72)	165 (48.10)	**0.0127**
Transverse colon resection	39 (3.90)	25 (3.80)	14 (4.08)	0.8640
Splenic flexure colon resection	60 (6)	38 (5.78)	22 (6.41)	0.6765
Left colectomy	52 (5.20)	33 (5.02)	19 (5.54)	0.7647
Sigmoidectomy	231 (23.10)	170 (25.87)	61 (17.78)	**0.0044**
Rectal resection	150 (15.0)	108 (16.43)	42 (12.44)	0.0928
Hartmann resection	24 (2.40)	7 (1.06)	17 (4.96)	**0.0003**
Abdomino-perineal resection	11 (1.10)	9 (1.38)	2 (0.58)	0.3484
Total colectomy	1 (0.10)	1 (0.15)	0 (0)	1.0000
Multiple resections	6 (0.60)	5 (0.76)	1 (0.29)	0.6702
Anastomosis, *n*. (%)				
Intracorporeal	799 (79.90)	525 (79.91)	274 (79.88)	0.9340
Extracorporeal	160 (16)	110 (16.74)	50 (14.58)	0.4139
Coloanal	6 (0.60)	6 (0.91)	0 (0)	0.0998
Operative time, min; mean ± SD	119.56 ± 36.30	120.39 ± 36.67	117.63 ± 35.45	0.7324
PO-ICU stay; *n*. (%)	17 (1.70)	5 (0.76)	12 (3.50)	**0.0030**
PO-ICU stay, days; mean ± SD	1.41 ± 0.79	1 ± 1	1.58 ± 0.90	0.2585
Time to discharge, days; mean ± SD	7.78 ± 5.60	7.56 ± 5.69	8.30 ± 5.36	**0.0467**
Clavien–Dindo; *n*. (%)				
No complication or CD I-II	947 (94.70)	624 (94.98)	323 (94.17)	0.6558
≥ III	53 (5.30)	33 (5.02)	20 (5.83)	
30-day mortality rate; *n*. (%)	11 (1.10)	4 (0.61)	7 (2.04)	0.0537

Bold values indicate statistical differences

*PO-ICU* Postoperative Intensive Care Unit; *ICU* Intensive Care Unit; *CD* Clavien–Dindo classification

Out of the 1000 patients, 947 (94.7%) experienced no complications or **CD** ≤ II, while only 53 patients (5.3%) experienced major complications.

The mean age of patients with no or mild complications was 65.60 years (± 10.61), whereas patients with severe complications were older (69.94 years ± 12.02, p = 0.0041). Gender distribution and BMI do not represent a risk factor for major complications (*p* = *0.2555*) (Tables 3 and 4).

**Table 3:** Univariate analysis of patients and surgical variables according to postoperative complications

	No complications or Clavien–Dindo ≤ II	Clavien–Dindo ≥ IIIa	*p* value
Number of patients; n. (%)	947 (94.70)	53 (5.3)	
Age, years;			
Mean ± SD	65.60 ± 10.61	69.94 ± 12.02	**0.0041**
Median (IQR)	71 (22–93)	71 (41–97)	
Female gender; n. (%)	424 (44.77)	19 (35.85)	0.2555
BMI; kg/m^2^;			
Mean ± SD	24.89 ± 4.54	25.60 ± 4.62	0.2686
Median (IQR)	24.21 (14.51–47.32)	24.82 (19.96–39.79)	
ASA classification; n. (%)			
I	81 (8.55)	2 (3.77)	**0.0001**
II	660 (69.70)	30 (56.61)	
III	204 (21.54)	18 (33.96)	
IV	2 (0.21)	3 (5.66)	
Comorbidities; n. (%)			
Cardiac disease	230 (24.29)	16 (30.19)	0.3283
Respiratory disease	22 (2.32)	0 (0)	0.6250
Other	121 (12.78)	9 (16.98)	0.3988
Multiple diseases	124 (13.09)	12 (22.64)	0.0616
Benignant disease; n. (%)			
Colon	91 (9.61)	4 (7.55)	0.8106
Rectum	0 (0)	0 (0)	
Malignant disease; n (%)			
Colon	737 (77.82)	39 (73.58)	0.2079
Rectum	119 (12.57)	10 (18.87)	
Stage for malignant disease; n (%)			
Stage 1	227 (23.97)	10 (18.86)	0.0985
Stage 2	314 (33.15)	19 (35.85)	
Stage 3	254 (26.92)	12 (22.64)	
Stage 4	61 (6.44)	8 (15.10)	
Previous abdominal surgery; *n*. (%)	130 (13.73)	18 (33.96)	**0.0004**
Previous chemotherapy; *n*. (%)	5 (0.53)	0 (0)	1.0000
Previous radiotherapy; *n*. (%)	2 (0.21)	0 (0)	1.0000
Previous radio-chemotherapy; *n*. (%)	25 (2.65)	2 (3.77)	0.6499

Bold values indicate statistical differences

*BMI* Body Mass Index; *ASA score* American Society of Anaesthesiologists score

**Table 4:** Analysis of intra- and post-operative outcomes according to **Clavien–Dindo** classification

	No complications or Clavien–Dindo ≤ II	Clavien–Dindo ≥ IIIa	*p* value
Number of patients; *n*. (%)	947 (94.70)	53 (5.30)	
Conversion to open surgery, *n*. (%)	23 (2.43)	0 (0)	1.0000
Procedures; *n*. (%)			
Right colectomy	398 (42.02)	28 (52.83)	0.1529
Transverse colon resection	39 (4.12)	0 (0)	0.2612
Splenic flexure colon resection	54 (5.70)	6 (11.32)	0.1254
Left colectomy	51 (5.38)	1 (1.89)	0.5172
Sigmoidectomy	226 (23.86)	5 (9.43)	**0.0120**
Rectal resection	140 (14.78)	10 (18.88)	0.4283
Hartmann resection	22 (2.32)	2 (3.77)	0.3668
Abdomino-perineal resection	11 (1.16)	0 (0)	1.0000
Total colectomy	0 (0)	1 (1.89)	0.0530
Multiple resections	6 (0.63)	0 (0)	1.0000
Anastomosis, *n*. (%)			
Intracorporeal	755 (79.72)	42 (79.24)	0.8626
Extracorporeal	153 (16.16)	7 (13.21)	0.7014
Coloanal	6 (0.63)	2 (3.77)	0.0629
Operative time, min; mean ± SD	119.03 ± 36.01	127.21 ± 39.97	0.1100
PO-ICU stay; *n*. (%)	15 (1.58)	2 (3.77)	0.2229
PO-ICU stay, days; mean ± SD	1.33 ± 0.72	2 ± 1.41	**0.0001**
Time to discharge, days; mean ± SD	7.29 ± 4.26	15.74 ± 13.58	**0.0001**
30-day mortality rate; *n*. (%)	0 (0)	11 (20.75)	**0.0001**

Bold values indicate statistical differences

A higher percentage of patients in the severe complication group were classified as ASA III or IV (*p* = *0.0001*).

**Fig. 1:**
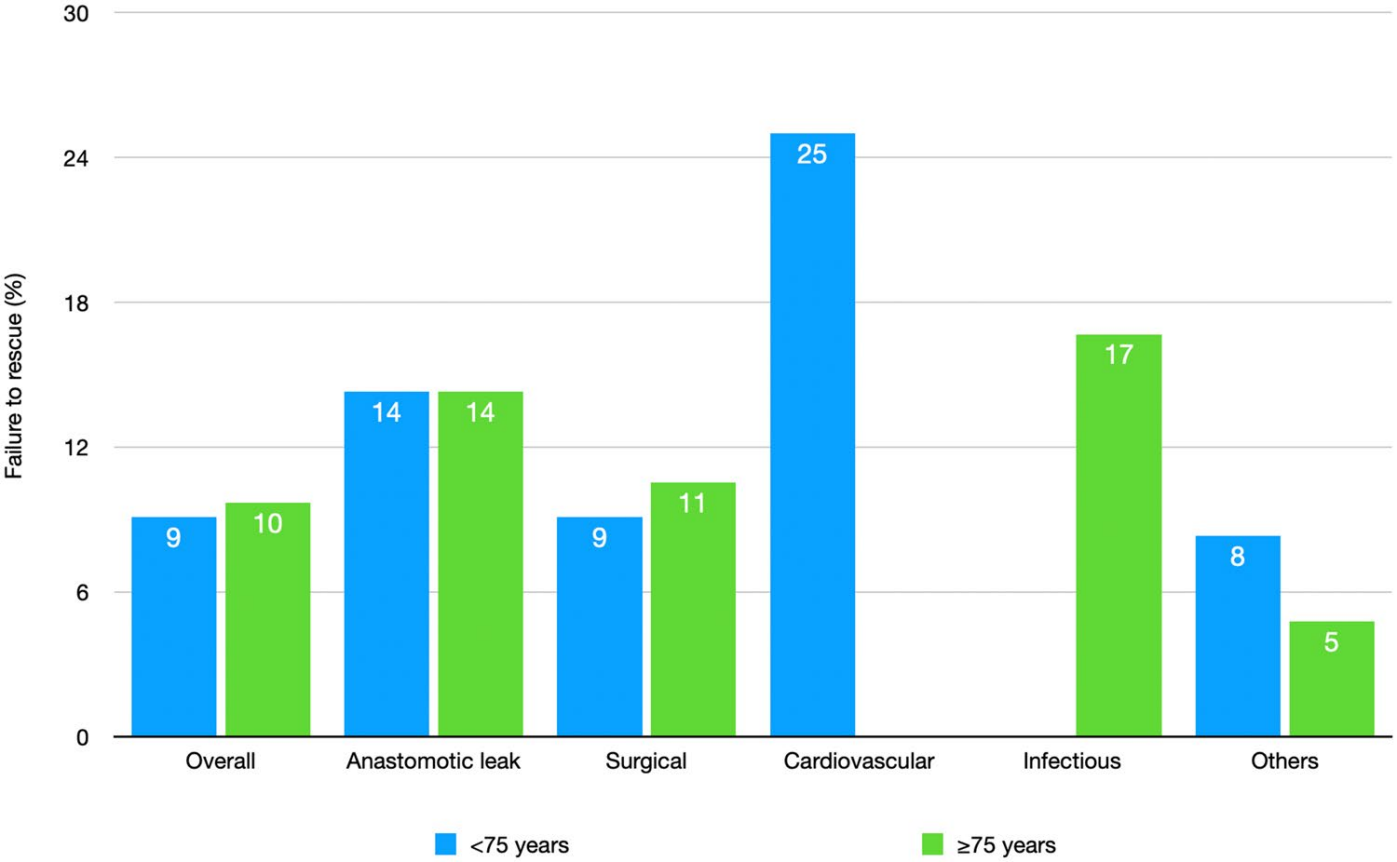
Bar chart comparing the failure to rescue (FTR) rates (%) in patients younger than 75 years (blue) and those aged 75 years or older (green) across different complication categories. The FTR metric refers to the proportion of patients who die following major postoperative complications. The overall FTR rate is comparable between the two age groups. Anastomotic leak-related FTR is identical for both age groups, standing at 14%, while surgical complications-related FTR is marginally higher in the elderly group (11%) compared to the younger group (9%). Cardiovascular complications-related FTR is significantly elevated in the younger group (25%). Conversely, infectious complications-related FTR is higher in the older population (17%) than in the younger group. *p* values are not statistically significative for all type of complications (colour figure online)

Figure 1 presents FTR rates across different types of complications: The overall FTR rate for patients under 75 years is 9%, while is slightly higher at 10% for elderly patients. No statistical differences were found between the 2 groups.

FTR rate for cardiovascular disease patients was lower in the elderly group (17% *vs.* 25%). This might suggest that younger patients who experience cardiovascular complications are at a higher relative risk of FTR, possibly due to the severity or unexpected nature of these complications in a younger population.

## Discussion

This study evaluated FTR rates in elderly patients (≥ 75 years) undergoing laparoscopic colorectal surgery compared to adults (< 75 years) in a high-volume Italian centre. Despite advances in surgical techniques and perioperative care, this analysis showed that elderly patients had higher FTR rates due to a greater risk of death following major postoperative complications.

A crucial finding was represented by the difference in 30-day mortality rates between the elderly and adult groups (2.04% *vs.* 0.61% respectively, *p* = *0.0537*), suggesting that age-related frailty plays a crucial role in determining outcomes after surgery. These data emphasize the key concept of tailored perioperative management strategies for frailty patients, especially in case of higher ASA score, reflecting poorer overall health status.

Frailty affects approximately half of older adults with cancer and is associated with an increased risk of adverse outcomes [21, 22, 48]. Integrating frailty assessment into cancer care pathways is essential for optimizing risk stratification and facilitating informed, shared decision-making, ensuring a personalized approach to treatment [49, 50]. This can prevent overtreatment in frail patients, mitigating the risk of severe functional decline and impaired quality of life, while allowing robust older adults to receive therapies that might otherwise be withheld based solely on chronologic age. Nevertheless, routine oncologic practice primarily relies on performance status rather than objective frailty assessment, despite evidence demonstrating the latter’s superior prognostic value and greater granularity in risk prediction [51, 52]. However, the lack of standardized frailty assessment leads to significant heterogeneity in its application, affecting both the proportion of patients classified as frail and the comparability of research data [52].

Interestingly, ERAS protocol implementation did not fully mitigate the risk of FTR in elderly patients, although it generally improved postoperative outcomes.

### Patients characteristics

Variations in FTR are determined by many patient factors like age, preoperative nutritional status, ASA score, cancer diagnosis, metastatic disease and emergency setting [8].

A crucial role is played by frailty, a common condition of decreased physiologic reserves in ≥ 75-year-old patients, consistently associated with higher FTR rates [27].

FTR incidence in elderly patients who underwent colorectal surgery changes significantly across studies, ranging from 5 to 20% [53–55].

Although this cohort showed no correlation between comorbidities and postoperative complications, many predictive factors were analyzed and evaluated in other literature experiences [56, 57].

Cardiovascular disease, chronic obstructive pulmonary disease (COPD), and diabetes represent the most common comorbidities that complicate postoperative course and, subsequently, impact FTR rates [56–60].

Patients over 75 years old represent 34.3% of the study population. They have a greater number of comorbidities compared to younger patients which determines a greater representation of stage 3 of the ASA classification; their neoplastic disease is more frequently localized to the colon rather than the rectum and many of them had already undergone at least one previous surgery and radio-chemotherapy. Elderly patients were more frequently subjected to Hartmann resections rather than sigmoidectomy with colorectal anastomosis, which are more frequent in younger patients. Furthermore, elderly patients needed intensive care more frequently and had a significantly longer length of stay.

Several studies showed that the main cause of death after major complications is pneumonia or respiratory failure [29, 61]. An interesting data that emerges from this study is the different types of complications in the two age groups. In fact, in younger patients, the complications are mainly cardiovascular while in elderly patients the most frequent complications are infectious ones, although statistical significance is not reached.

Concerning emergency surgery, as reported in the literature, bowel obstruction or perforation double FTR when compared to elective procedures [62].

In this series, patients who experienced FTR had longer hospital stays and increased readmission rates.

Some studies have evaluated FTR and mortality in colorectal surgery over time. Fry et al. compared FTR and mortality data during 2005–2006 with those during 2013–2014. They observed a reduction in FTR from 25.2 to 22.7% and a subsequent reduction in mortality from 9.7 to 8.7% [63].

### Open vs. minimally invasive approaches

In the last decades, minimally invasive surgery advent has significantly changed the landscape of colorectal surgery [64–66]. In addition, new anesthesiologic techniques were used to prevent postoperative complications, especially in elderly patients [67, 68].

Several studies showed lower FTR rates in patients who underwent laparoscopic surgery compared to open surgery due to fewer postoperative complications including surgical site infections, shorter hospital stays, and quicker recovery times [9, 69–71].

Patel et al*.* reviewed data collected between 2005 and 2018 of adult patients undergoing emergent colectomy. The authors performed a Propensity Score Matching based on the demographic and comorbidity data of patients who underwent laparoscopic and open surgery. After matching, 11,484 cases were included for analysis, of which 3829 were laparoscopic. After an extensive analysis, Patel et al. concluded that open surgery significantly increased the risk of nearly all measured postoperative complications including return to operating room, ventilator use > 48 h, and septic shock.

Concerning FTR after robotic colorectal surgery, no data are available in the literature to date.

### Hospital facilities

The findings of this study indicate that hospitals with well-established rapid response teams and standardized complication management protocols exhibit lower FTR rates.

Colorectal surgery is associated with an elevated risk of comorbidities [37, 61, 72–75]. In particular, the overall complication rate after laparoscopic colorectal surgery is more than 15% [64].

In colorectal surgery, the volume of activity is one of the characteristics of a measurable process that can have a significant impact on the outcome of health care: hospitals performing a higher number of procedures tend to achieve better outcomes, including reduced mortality rates, fewer complications, shorter hospital stays, and lower overall costs, along with improved patient prognosis [76–78]. For colon cancer, a minimum annual caseload of 50 procedures is recommended, as increased surgical volume is associated with a progressive decline in 30-day mortality rates. In the case of rectal cancer, which involves greater technical complexity and demands a more sophisticated multidisciplinary approach, the suggested threshold is around 15 cases per year. Despite these findings, low-volume were represented by hospitals that performed < 40 colorectal cases/year, intermediate-volume from 40 to 64 cases/year, and high-volume ≥ 65 cases/year [77].

According to Ruffo et al. [79], our hospital represents a tertiary referral center.

However, hospitals with the same incidence of complications experience different mortality rates [2, 29, 30]. According to Silber et al*.*, the incidence of complications is associated primarily to patient characteristics, while mortality rate is related to both hospital and patient characteristics [30]. Following these findings, in 1992, Silber et al*.* introduced the concept of FTR as the ability to recognize and treat postoperative complications and the capability to reduce inpatient mortality [30]. This parameter is associated more with hospital characteristics and less with patients [30].

Consequently, Henneman et al*.* have shown that the most important hospital factors that influence FTR are the hospital volume, hospital type and hospital ICU level [55].

Several studies found that low-volume hospitals had markedly higher FTR compared with higher-volume hospitals (30.3% *vs.* 13.1%) [31, 80], but this association seems significant for those surgeries considered more challenging than colorectal ones [31, 55, 80]. Moreover, the FTR is significantly lower in university hospitals and in teaching hospitals than in non-teaching ones [31, 63, 81].

Similar data are reported in the literature, suggesting that experience and resources available at high-volume centers play a crucial role in managing complications, providing lower FTR rates when compared to low-volume hospitals [82–84].

Maybe, these results are achieved thanks to better systems for early identification and intervention in case of postoperative complications by high-volume hospitals. These skills are guaranteed by structured team-based approaches and standardized care protocols, to enhance patient surveillance and intervention.

Concerning ICU level, it impacts independently FTR: ICU levels 2 and 3 achieve lower FTR rates when compared to level 1 [31].

Our findings are comparable to those of other centers with the same volume of cases/year, and the same teaching and ICU levels.

### Limitations

Although this study involves more patients than previous studies, it still has some limitations. First, owing to the study’s retrospective nature, potential bias may exist. However, all the one thousand patients were operated by the same surgical team, using the same techniques and technologies, so eliminating a potential bias in surgical treatment.

A major limitation of this study is the bias introduced by homogenizing colonic and rectal cancer. These malignancies differ in tumor biology, treatment strategies, and surgical complexity. Rectal cancer often requires preoperative chemoradiotherapy and TME, increasing surgical risks and influencing outcomes differently from colonic cancer, which is typically managed with upfront surgery. Combining these entities may introduce confounding factors, affecting the interpretation of surgical feasibility and oncologic results. Future studies should analyze colonic and rectal cancers separately to improve the accuracy of prognostic evaluations and better assess procedure-specific risks and outcomes.

## Conclusions

Elderly patients have a greater number of comorbidities, need intensive care more frequently and have a significantly longer length of stay compared to younger ones. This suggests that age-related frailty plays a crucial role in determining outcomes after surgery. However, FTR differences are not significant between adult and elderly patients. The findings of this study indicate that a minimally invasive approach, skilled team, well-established rapid response and standardized complication management protocols can have a positive impact on FTR regardless of the patients age.

## What does this paper add to the literature?

Identification of baseline characteristics of patients and intra- and postoperative data linked to higher failure to rescue in adult and elderly populations who underwent laparoscopic colorectal surgery in a referral centre.

## Data Availability

The raw data supporting the conclusions of this article will be made available by the authors, with undue reservation.
